# The Healing Power

**Published:** 1886-09

**Authors:** 


### *THE HEALING POWER.*

Ours, we say, is the age of intellect, and mind holds the reins over the fast flying steeds of material progress. Even in the realm of bodily disease there is now a more general belief than ever before that "As a man thinketh in his heart so is he."

Coleridge says: "In the treatment of nervous diseases he is the best physician who is the most ingenious inspirer of hope."

Dr. Oswald, reversing the proposition, declares that "lost hope is a fatal though silent disease."

"Pain," says the *Scientific American*, "is chiefly mental, and the severity of it would be comparatively slight, did we not give ourselves up to it. Animals suffer less than men. A horse will feed after breaking a leg, while a man would lie and moan."

From innumerable sources we find emphasis of the idea that the spirit controls the body. "To retain or recover health," says one writer, "persons should be relieved from anxiety concerning disease. The mind has power over the body—for a person to think he has a disease will often produce that disease. This we see effected when the mind is intensely concentrated upon the disease of another. We have seen a person seasick, in anticipation of a voyage, before reaching the vessel. People have died of cancer in the stomach, when they had no cancer in the stomach or any other mortal disease. Therefore persons should have their minds diverted as much as possible from themselves. If he wills not to die, a man can often live in spite of disease; and, if he has little or no attachment to life, he will slip away as easily as a child will fall asleep. Men live by their minds as well as by their bodies. Their bodies have no life of themselves; they are only receptacles of life—tenements for their minds—and the will has much to do in continuing the physical occupancy or giving it up."

In a pamphlet recently published, we find the following statements which no fair-minded person will question: Many chronic invalids are simply the victims of a chronic mode of thought; they have formed the *habit of being sick*, and they could if they would, or rather if they knew how, form the habit of being well. So many believe that they cannot help being weak, nervous, ailing, and miserable, and they live year after year bound with the fetters which they have forged for themselves. Many a woman frets herself sick, and many a man has lost his life from an overtaxed mind, which has brought corresponding disease to the body.

"How often does it happen that a physician who has practiced a specialty for a number of years becomes at last a victim to the very disease which he has labored so long to overcome in others. It has been a picture before his mental vision which at last finds outward expression upon his body."

The Rev. C. E. Mann, in the *New Church Messenger*, writes in inspired strain on the " Healing of the Body through the Soul." We have space only for brief extracts.

" Apart from spirit, man's body is an utterly powerless collection of substances that will quickly be resolved into their original elements. The body has no power to be anything or to do anything, except it be given of the soul. How transcendently important it is, then, if we would be blessed with that healing of the body which the powers of spirtual life can bring us, that we recognize the reality and the power of spiritual things; that we refuse to give material forces control over us by believing in them; that we come under the dominion of the great flowing river of life and of strength which comes down through heaven and the spirtual world into our souls from the Lord. The very existence of the material universe is as dependent upon the spiritual universe as the shadow is dependent upon the light that casts it. And yet we turn our backs upon the infinite power and reality within, and resolutely fix our eyes upon the shadow, giving it our allegiance, having faith in it, trusting it, and worshiping it; thus by the very action of our thoughts in reference to it, closing our souls to the current of the descending actual life from above.

" We must, then, if we would realize the true benefit in the health of our bodies which can come from the spiritual power of heaven, believe in that power. We must accept the doctrine that in spiritual life is all true life, that in spiritual power is the only power, and that in spiritual reality is the only substance.

" If in your conversation you treat the sicknesses with which you are afflicted as though they were really enemies of your body, possessing an actual self-existent nature, you thereby give them a hold upon you which they would otherwise not possess. To be continually looking into the ill feelings of your body, always examining your pulse, as it were, forever thinking of this ache or that discomfort, analyzing all the strange or peculiar sensations that may come to you, and discussing such matters with others—all such conduct gives a basis in your mind for the presence of evil thoughts to aggravate and maintain your ailments. Such methods of thought and conversation are among the things that most do hinder the presence and the efficiency of the health-giving influx of heaven.

" Yet it seems as if the dominant habits of polite society made such topics as these the leading subjects of anxious inquiry and discussion. It is the very method in the law of spiritual doctrine that would most successfully fasten disease upon us, and it makes the spiritual cure of our sicknesses impossible.

"Let our association with each other be healthful. All our conversation, thoughts we cherish, the sphere of our affections, should go forth to help all with whom we are associated, to strengthen them against sickness, to weaken the hold of the disease-producing influences upon them. Rather live in your health and turn your back upon your sickness, and be not anxious as to the result. Anxiety and fears open the doors for the admission of disease."—*Laws of Life.*

